# Upstream Ecological Risks for Overweight and Obesity Among African American Youth in a Rural Town in the Deep South, 2007

**Published:** 2010-12-15

**Authors:** Alison J. Scott, Rebecca F. Wilson

**Affiliations:** Jiann-Ping Hsu College of Public Health, Georgia Southern University; Georgia Southern University, Statesboro, Georgia. Ms Wilson is affiliated with the US Department of Health and Human Services, Health Resources and Services Administration

## Abstract

**Introduction:**

Few studies have focused on overweight and obesity among rural African American youth in the Deep South, despite disproportionately high rates in this group. In addition, few studies have been conducted to elucidate how these disparities are created and perpetuated within rural communities in this region. This descriptive study explores community-based risks for overweight and obesity among African American youth in a rural town in the Deep South.

**Methods:**

We used ecological theory in conjunction with embodiment theory to explore how upstream ecological factors may contribute to risk of overweight and obesity for African American youth in a rural town in the Deep South. We conducted and analyzed in-depth interviews with African American community members who interact with youth in varying contexts (home, school, church, community).

**Results:**

Participants most commonly stated that race relations, poverty, and the built environment were barriers to maintaining a healthy weight.

**Conclusion:**

Findings suggested the need for rural, community-based interventions that target obesity at multiple ecological levels and incorporate issues related to race, poverty, and the built environment. More research is needed to determine how disparities in obesity are created and perpetuated in specific community contexts.

## Introduction

Rural areas in the Deep South have a disproportionately high prevalence of obesity compared with urban areas of the United States (1-3), as have African American populations compared with white American populations (4). Almost one-third of youth aged 2 to 19 years are categorized as overweight or obese (5), and rural youth are more likely to be obese than urban youth (6,7). However, little research has focused on the problem among rural African American youth in the Deep South. In addition, few studies have explored how these disparities are created and perpetuated in specific rural communities.

Ecological theory, which is increasingly used to inform public health research and intervention (8,9), can be applied to complex community contexts and to the exploration of links between social structures, individual behaviors, and health. Developed by Sweat and Denison (10), this multilevel approach assesses the interplay of *downstream* factors at the individual level (such as knowledge and attitudes) and the relational level (such as family support) with *upstream* factors in influencing health. Upstream levels incorporate environmental factors (such as the built environment), structural factors (laws and policies), and superstructural factors (social justice issues such as race and class).

Research that examines how upstream levels of the ecological theory, including superstructural factors such as racism and poverty, may contribute to disparities in rural risk for overweight and obesity is scarce. We coupled the ecological theory with embodiment theory, which argues that "bodies tell stories . . . about the conditions of our existence" (11). Embodiment theory asks how the contexts of our lives are reflected in the health of our bodies. When used together, these frameworks help to explore how social factors affect health risks. We explored upstream ecological factors that may facilitate development of obesity or serve as barriers to maintaining a healthy weight among African American youth in a rural community in the Deep South.

## Methods

This study was conducted as part of a larger qualitative community health assessment in a county in rural southeastern Georgia (12). Researchers worked with a citizens' health collaborative to conduct the study. The institutional review board at Georgia Southern University reviewed the study, and all participants provided written informed consent. The study community was a rural town in southeastern Georgia. Roughly 40% of its 2,200 residents are African American, and 28% are younger than 18 years. The median household income is less than $21,000, and more than half of families with children in the community live below the federal poverty level ($17,463 for a family of 4 in 2000) (13).

Researchers conducted in-depth interviews (Appendix) with 18 African American community members ranging in age from 24 to 70 years. Participants were purposefully sampled to select those who interacted with youth in varying contexts (home, school, church, socially) to incorporate a range of perspectives. Tabular summaries of participant characteristics helped to maximize variability in sampling. A key informant, the director of the local health department, suggested initial participants; participants were later identified through snowball sampling and through referrals from a range of community organizations.

All participants were African Americans who were longtime residents of the community and who interacted with African American youth. They included parents, grandparents, a school board member, teachers, a principal, pastors, the high school band director, the coordinator of a church-based youth resource center, and the president of the local National Association for the Advancement of Colored People, among others. Interviews were conducted between January and April 2007.

Interviews were conducted in person in the home or community setting of the participant's choice and lasted from 20 minutes to 2 hours. Interview guides were used to conduct the interviews. Questions addressed the 3 upstream levels in Sweat and Denison's ecological model (Appendix). At the environmental level, topics included the community-built environment and the food environment. Questions included, "Where do youth go to get exercise?" and "Where do families eat when they eat out?" At the structural level, topics included city governance and community organizations. Participants were asked questions such as "How inclusive is community leadership?" and "What organizations are key for youth?" At the superstructural level, topics included race and class relations. For example, questions included, "Tell me about race relations in the community" and "Where do people get jobs here?" Interviews were recorded and transcribed. Content analysis of interview transcripts and field notes was ongoing during data collection. Transcripts were coded to identify text segments related to each ecological category, which were grouped together for analysis by category (14). Themes were identified and expanded by using reflexive memo-writing (15) and through data display in matrices (16). Ideas that emerged from content analysis were triangulated by having both researchers involved in analysis and through member checking by sharing results with community coalition members (17).

## Results

Analysis of transcripts revealed that many participants perceived that although racism was more subtle than in times past, it was still rampant in their community and was a stressor for youth independently and in interaction with socioeconomic status (SES). In addition, issues emerged related to the built environment, including lack of access to healthful food options and venues for physical activity; these issues, in turn, were reported by participants as interacting with racism and low SES.

### Black and white: race relations as a stressor

Participants described their community as *de facto* segregated by race; most African Americans live on the south side of town and most whites live on the north side of town. The community's churches and civic organizations are also mainly segregated. As a case in point, the citizens' health collaborative, which was composed of leaders from local businesses, schools, health care facilities, and city government, requested the larger community health assessment. The collaborative had no African American members. A former school board member, the sole African American representative on the school board at the time, commented on the racial dynamics:

You stay on your side of the tracks. . . . Change is slow. . . . It's black and white, black and white.

A young woman agreed, commenting that white people came to her neighborhood only to buy drugs:

If you see a white person driving through on this street, it's mostly for drugs. Like, white people don't just come through here.

This spatial segregation, according to participants, limited the interaction of black and white community members.

The public schools, however, being integrated by law, served as a lightning rod for racial tensions, according to participants. The middle school and high school have been the settings of major racial incidents in recent years concerning issues such as the practice of having separate black and white prom courts, student councils, and class presidents; outcry over the lack of African American teachers; and a lawsuit challenging an ability grouping curriculum. (The US Office of Civil Rights ruled that the curriculum was discriminatory.) As such, African American youth have been, and continue to be, at the center of the community's struggle with race issues. In addition, such experiences of segregation and racism are carried into adulthood and, at times, out of the community. A recent alumnus of the local high school described going to college and being figuratively "slapped in the face" by other African American students who were appalled at this community dynamic:

Those kids [at college] who came from bigger schools in North Georgia or other states said, "Don't you all know that you all are still segregated?" . . . That was a slap in the face to us, and we had to hide that here in our hearts. . . . We're so far behind. . . . It's scary.

Although one cannot assume this situation translates into race-related stress for every African American student, such a community context creates ideal conditions for such stress.

### Without a dream: entrenched low SES as a stressor

Racial issues were intertwined deeply with those of class in the community. In addition to the strain of racial tension, many African American youth in the community endure the stresses of poverty and face a future with limited opportunities to escape poverty as adults. Participants consistently reported that job options were few in the depressed local economy, except for fast-food jobs, other minimum-wage service jobs, and a poultry processing plant. The community has undergone economic deterioration in the past 30 to 40 years. Farming work was lost as agriculture became more mechanized and local textile factories closed or relocated. In addition, many participants shared that as the local economy weakened, illegal drug activity had exploded, providing a lucrative yet potentially destructive vocation for African American youth. A pastor saw this choice as an inevitable consequence of the economic realities faced by black youth as they enter adulthood:

What you gonna do when you graduate from high school? What jobs are available? Outside of going to work in the poultry plant, working at a fast-food place, what else is there to do? . . . The big business here is drugs and eventually jail.

In addition, there was consensus that race and nepotism entered into the struggle to find work, which fed the sense of hopelessness among black adolescents. This was described as the *who-you-know syndrome* or the *old-buddy syndrome.* Because white community members owned most local businesses, it was claimed that they controlled access to the best jobs in town; those who are hired have the right social connections, it was purported, even if they do not have the right skills:

That's the status quo of every . . . top position here right now. . . . [It depends on] who you know, your great-grandfather. They're not qualified on paper. They don't know a hill of beans about the job. . . . They just got it handed down. This is not fair to the blacks in the community. . . . We're so far behind it's not even funny, and it's killing us.

The African American youth, facing barriers and frustrations related to both race and class, were characterized by an African American pastor who worked with them as lacking in hope and trust, and as "people without a dream.*"* A mother lamented, of African American young men especially, that *"*they get lost*"* as soon as they enter high school.

### Nowhere to go: the built environment

Issues related to the built environment were frequently voiced by participants. A common complaint expressed by parents, teachers, counselors, and community leaders was the lack of accessible venues for youth to be active. As put by a former teacher:

There's nowhere for the youth to go, nothing to do. . . . There's not one skating rink. There's not one bowling alley . . . [to] let them exert energy.

The town is home to a central park. However, in recent years the park has closed at 6:30 p.m., limiting its availability after school. The early closure, which was sparked by illegal drug activity in the park, blocks access to basketball courts and playing fields. The community has a recreational center that sponsors sports activities. However, participants commented that activities had a participation fee and were geared toward younger children. In addition, this recreational center was constructed several miles west of the town center in an undeveloped area, off a 2-lane highway with no sidewalks, making transportation an issue for many families. A middle school principal summarized:

We really don't have that thing to do with our middle school-aged kids and our high school students. . . . [We need] something positive in the community. Not just something for blacks. Not just something for whites. . . . We don't have anything like that.

Multiple participants discussed youth activities sponsored by churches that attempted to fill the gap. However, activities often were short-lived, underfunded, and available only to youth from the church's congregation. Participants complained that this fed a divisiveness within the African American community that hampered efforts to address the problem. A mother commented:

It's about 99,000 different churches, and all of 'em have their own little center, but it's basically just for those kids that go to their church. It's just got no togetherness.

The community also has limited options for accessing healthful foods. The town has 1 grocery store and convenience stores that sell packaged food. With the lack of competition, the grocery store "can set . . . its own prices" and has limited selections of produce and other healthy, fresh foods. Restaurant options largely include fast-food chain restaurants and buffets serving a traditional array of energy-dense, high-fat foods. In addition, these restaurants provide the most obvious source of employment for local youth. Many employees are provided discounted or free food. One mother described how her daughter, who has type 2 diabetes, had gained weight after she started working at Hardee's:

It was when she started working at Hardee's. She would just bring a sandwich home and eat it at night . . . and all of a sudden she picked up all this weight, and we went to the doctor. . . . I don't want her to have to take insulin — not this young.

### Intertwined: the built environment, race, and poverty

Findings suggested that issues related to the built environment interacted with issues of race and class. For example, for access to more food options, residents with transportation and money for gas could drive 20 miles to a Wal-Mart or a Save-a-Lot in a neighboring county. Those without such resources had to rely on local grocery options, given the lack of public transportation. A disproportionate number of African American families are low-income, placing them in the latter situation.

In addition, participants were aware of additional pay-to-participate recreational opportunities for youth to be active. A mother said, "There are more programs the whites can get into because their mama and daddy got more money than the rest of us." As for the community's recreational center, some participants perceived racial bias in the decision to place it outside of town and to charge fees, which they linked to the overrepresentation of middle- and upper-class white children in the programs. A pastor asserted:

We do have a recreational authority and all that good stuff, but it's not equal. It's racially biased. When you look at the programs and who's actually involved, [there aren't] enough blacks in proportion to whites. I don't mind going on the record saying this. That's the way it is.

## Discussion

Study findings indicated that stressors associated with racism and low SES are likely daily occurrences for African American youth in this context. Such environmental stressors have been shown to be associated with increased activation of physiologic stress pathways, including the hypothalamic-pituitary-adrenal (HPA) axis; chronic HPA stimulation can lead to metabolic disruption that may contribute to increased risk of obesity (18,19). These physiologic pathways provide a biological framework for embodiment theory; external stresses related to race and poverty can enter the body and be reflected in dysregulation and eventually disease. Epidemiologic studies bear this out. For example, the Black Women's Health Study, a prospective study of 43,000 black women, found a significant association between weight gain and levels of both everyday racism (experienced daily) and lifetime racism (accumulated experiences), leading the authors to postulate that racism may contribute to racial disparities in obesity in African American women (20). Other studies have identified associations between high perceived stress and increased production of cortisol, which may lead to metabolic dysregulation and overweight (21,22). Ethnographic data from other studies of rural communities show race, poverty, and lacking the right social connections to be sources of strain in rural towns from Appalachia to the Mississippi Delta (23).

The importance to diet and physical activity of the physical surroundings, the built environment of communities, has been established (24,25). In this rural community, the local built environment emerged as another potentially salient theme, in the ways it restricts youth access to healthful food options and physical activity. In concordance with this finding, Larson, Story, and Nelson's 2009 review of 54 studies reported that rural, low-income, and minority neighborhoods tended to have poor access to supermarkets and healthful foods (26). In addition, Bove and Olson identified lack of transportation and rural isolation as barriers to healthy eating in rural New York State (27).

As described by participants, issues related to the built environment interacted with issues of race and class in this rural community. Fees and location were barriers to poor and African American children using recreational facilities; in addition, time and individual transportation were needed to access better food options. Beyond co-localizing unhealthy built environments with poor and minority neighborhoods, most studies do not address the interplay of the 3.

These study findings may be transferable to other rural community settings that have health disparities related to race and SES. Further work is needed that involves children and youth directly and that integrates mixed methods and measures, including metabolic stress indicators. This study is descriptive and exploratory and is not intended to test causal relationships among the built environment, racism, SES, and overweight and obesity. However, research of this kind is needed to illuminate how disparities in overweight and obesity may be created and perpetuated in specific community contexts. A study limitation is the lack of inclusion of the local prison populace.

The findings highlight the need for rural community-based interventions that target overweight and obesity at multiple ecological levels, including interventions targeting embedded racism and environmental barriers to poor people in accessing healthy foods and physical activity opportunities. The findings also illustrate the value of community-based research that elucidates how race, class, and environment become embodied as overweight and obesity in specific community contexts.

## Appendix

### Interview guide: upstream ecological risks for overweight and obesity among African American youth in a rural town in the Deep South, 2007

Below is a list of topics addressed in in-depth interviews with participants. Questions listed were not necessarily asked verbatim or in order, and different topics were emphasized in different interviews, depending on the participant. Other topics were added in response to the input of the participants. This emergent approach to data collection ensured that the research explored issues that are most salient to participants.

1. Introductions and description of study
2. Consent process
3. Can you tell me a little bit about yourself?
  Probes: family, work, how interact with youth.
4. How long have you lived here?
5. Can you tell me about the community here?
  Probes: Are you happy here? Do you feel safe? What community groups and organizations are you part of? What are the major challenges the community faces with regard to health, jobs, education?
6. Environment
  - Eating
    Probes: Where do people eat out? What grocery stores and restaurants do you have? Where else do you get food, etc?
  - Exercise
    Probes: What do youth and families do for fun? Where do youth go to get exercise? What venues are there for getting out? Do the schools require gym?
  - Do you think your community is built in a way to encourage people to be active?
    Probes: What about sidewalks? Parks? Sprawl? How is new development occurring?
7. Structural level
  - Governance
    Tell me about your city and county government and officials (city council, mayor, county commissioner).
    Probes: Are they effective? How inclusive? Major policies? Turnover? How related to development?
  - Community organizations and institutions
    What are the major organizations in town? What role in eating and exercise?
    Probes: Churches, schools, civic groups, clubs.
8. Race and class
  - Race
    Tell me about race relations in the community.
    Probes: History? Housing? Schools? Jobs? Segregation?
  - Class
    How well off are families financially in the community?
    Probes: Where do you get jobs? TANF (Temporary Assistance for Needy Families)? College? Moving away?
9. Other
10. What questions can I answer for you about what we're doing?
11. Sum up and debrief. Thank you.
